# Modelling Causal Factors of Unintentional Electromagnetic Emanations Compromising Information Technology Equipment Security †

**DOI:** 10.3390/s22187064

**Published:** 2022-09-18

**Authors:** Maxwell Martin, Funlade Sunmola, David Lauder

**Affiliations:** School of Physics, Engineering and Computer Science, University of Hertfordshire, Hatfield AL10 9AB, UK

**Keywords:** compromising emanations, TEMPEST, vulnerability likelihood, causal factors, interpretive structural modelling, fishbone diagram

## Abstract

Information technology equipment (ITE) processing sensitive information can have its security compromised by unintentional electromagnetic radiation. Appropriately assessing likelihood of a potential compromise relies on radio frequency (RF) engineering expertise—specifically, requiring knowledge of the associated causal factors and their interrelationships. Several factors that can cause unintentional electromagnetic emanations that can lead to the compromise of ITE have been found in the literature. This paper confirms the list of causal factors reported in previous work, categorizes the factors as belonging to threat, vulnerability, or impact, and develops an interpretive structural model of the vulnerability factors. A participatory modelling approach was used consisting of focus groups of RF engineers. The resulting hierarchical structural model shows the relationships between factors and illustrates their relative significance. The paper concludes that the resulting model can motivate a deeper understanding of the structural relationship of the factors that can be incorporated in the RF engineers’ assessment process. Areas of future work are suggested.

## 1. Introduction

Electronic products generate electromagnetic interference and can also be susceptible to it. Electromagnetic compatibility (EMC) standards require electronic products not to generate unacceptable levels of interference, but also to have an adequate level of immunity from them [1]. From an information security perspective these support the security objectives of integrity and availability. That is, the equipment being interfered with will continue to function and operate correctly. However, reducing emissions so that products can coexist in the same environment does not necessarily protect the security objective of confidentiality. Where information technology equipment (ITE) is processing sensitive information, the electromagnetic fields that it generates can give rise to unintentional emanations that can radiate into space or conduct along power and signal lines. These emanations, if related to the information being processed, can be captured and reconstituted, leading to a loss of confidentiality. The name given to describe these vulnerabilities was TEMPEST [2].

TEMPEST vulnerabilities of office-based ITE (computers and peripherals) were demonstrated publicly by [3]. This showed how unintentional emanations could be captured and reconstructed from a visual display unit (VDU), resulting in a loss of confidentiality. The author of [3] established that the emanations were either narrow-bandwidth (clocks and their harmonics) or wide-bandwidth (video signals). It also made the distinction between EMC and TEMPEST, showing that ITE meeting EMC radiation and conduction standards can still have its security compromised.

As ITE has developed and been tested against EMC standards, research continues to show ITE exhibiting TEMPEST vulnerabilities, e.g., liquid crystal display (LCD) monitors [4], wired and wireless keyboards [5] and touch screens [6]. This, along with claims that radiated emissions can be recovered up to 200–300 m away, and that conducted emissions travel many kilometers [7], indicates that these vulnerabilities are still prevalent. Research has also focused on the mechanisms producing TEMPEST vulnerabilities and their mitigations. This has shown how electronic printed circuit boards (PCBs) and their components act as antennas, but also how redesigning circuit board layouts, decoupling and filtering signals and installing electromagnetic shielding at the equipment, system, room, building and facility levels can help reduce the effects [8].

An outcome of this research is the recognition that TEMPEST vulnerabilities need to be considered in the design, manufacture, remanufacturing, testing and quality assurance of systems and processes. Quality control during manufacture is assured by certifying TEMPEST-approved equipment against international standards [9], as even minor differences in manufacturing quality can lead to unintentional emanations. This enhanced quality control and assurance is perceived to increase costs, driving project procurement decisions towards commercial off-the-shelf (COTS) equipment. This has security implications, as COTS equipment will meet EMC emission limits but could still be leaking sensitive information.

The comprehensive list of 26 causal factors previously identified when considering the TEMPEST vulnerabilities of office-based ITE [10] may have wider applicability to other scenarios, such as where communication and encryption equipment have been deployed, either as standalone devices or embedded in computing equipment. Furthermore, it is estimated that there will be ~30 billion networked devices by 2023, with ~15 billion of them being machine-to-machine (commonly referred to as the Internet of Things (IOT)) devices [11]. This raises some significant cybersecurity challenges, as many of these devices will use the same Internet protocols to connect, and will therefore be open to new and established system and network attacks. It is predicted that the number of network distributed denial of service (DDoS) attacks will grow to ~15 million by 2023, a doubling since 2018 [11]. One of the key cybersecurity concerns of IOT endpoint devices (sensors and actuators) is their authentication into the wider network. Wireless sensor networks, vehicle communications and wearable and medical devices, along with cyberphysical human systems, employ authentication mechanisms with different levels of sophistication depending on the resources at their disposal, e.g., a sensor to measure a physical quantity may have limited resources in terms of electronics and power [12]. This has implications in terms of attack vectors. For example, it may be easier to attack certain IOT devices because they are easier to gain physical access to. Others may also have TEMPEST vulnerabilities, which could provide the information needed by attackers to access networks from endpoints.

Technological development and its expanding usage not only increase the potential for vulnerabilities but also the opportunities for potential attackers. The availability of low-cost receivers (e.g., software-defined radio (SDR)), antennas and signal processing software now make TEMPEST vulnerabilities a more attractive target. Consequently, TEMPEST vulnerabilities, like other cyber vulnerabilities, need to be addressed as part of a risk-management approach [13,14]. To risk-manage an organization’s information assets requires knowledge of the potential threats and their capabilities and the vulnerabilities of the organizations people, processes and technology, as well as the detrimental impacts should their information or systems used to process it be compromised [15]. Without an understanding of the causal factors, their interactions and relationships that give rise to compromising unintentional emanations, it can be difficult for cybersecurity practitioners without appropriate and relevant RF experience to assess the severity of these vulnerabilities within the wider cyber vulnerability context. In practice, this means there is a reliance on radio frequency (RF) expertise to quantify likelihood of exploitation [16].

Whilst previous studies have identified several causal factors, there remains a knowledge gap as to their structural relationships and their relative significance when performing a vulnerability assessment on office-based ITE. Consequently, the aims of this paper are to:

1. Explore how RF engineers can categorize the known causal factors as belonging to threat, vulnerability, or impact.
2. Model the vulnerability causal factors so that their interpretive structure, relationships, and relative significance can be understood and shared with cyber- and information security professionals without RF experience.

Categorization of the causal factors facilitates the modelling of the interpretive structure of the causal vulnerability factors. A participatory modelling approach is adopted in this paper. It involves the use of experienced RF engineers, with a view to answering the following research questions:

RQ1: What is the interpretive structural relationship between the identified causal vulnerability factors?
RQ2: What is the relative significance of the causal factors that give rise to unintentional electromagnetic emanation vulnerabilities?

The remaining sections of this paper are structured as follows. Section 2 details the research methodology, consisting of the use of RF engineer focus groups, cause-and-effect analysis and the interpretive structural modelling technique applied to the vulnerability factors. Section 3 contains the results, consisting of the categorized list of causal factors and the structural model. Section 4 discusses the results obtained. Conclusions and areas of future work are suggested in Section 5.

## 2. Methodology

**Figure 1:**
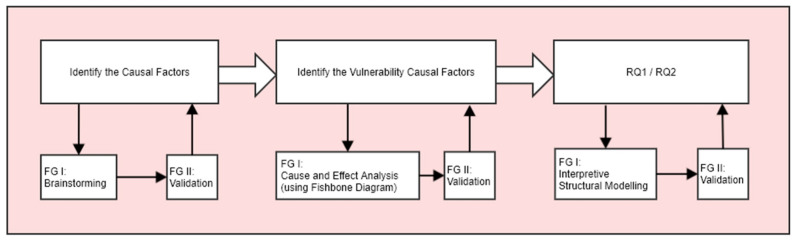
Use of focus groups to address the research questions.

The focus of this study is the loss of confidentiality through unintentional emanations. The risk of this is a function of threat, vulnerability and impact [15]. To answer the research questions RQ1 and RQ2 stated in Section 1, the research methodology adopted is shown in Figure 1. Central to this approach is the use of focus groups consisting of experienced RF engineers.

Two focus groups from different organizations, FG-I and FG-II, were engaged in this work. The FG-I focus group was presented with a list of the causal vulnerability factors and asked to create the ISM model from them. The other focus group, i.e., FG-II, was used to validate the structural model obtained from FG-I. The focus group participants were over 18 years of age and could (as assessed by the team leaders in their respective organization’s) apply their RF engineering skill to unintentional electromagnetic emanation-related problems.

### 2.1. Identifying the Vulnerability Causal Factors

In the previous study [10], a workshop was held with focus group FG-I. They were asked to brainstorm the factors that would lead to the compromise of information, resulting from unintentional emanations, from an office-based (thin client workstations) ITE scenario. Following the workshop, the seven RF engineers in FG-I were asked to reflect on the list of brainstormed factors and suggest any additions or modifications. Changes to the list were admissible where the majority (four or more) agreed. After two iterations, the list of causal factors with their rationale for inclusion was agreed. The four RF engineers in FG-II were then asked to validate the list of factors, by confirming that the list was complete and that the reason for a factor’s inclusion was sound. Finally, both focus groups were engaged at multiple workshops to agree the final list of factors along with the rationale for the factor’s inclusion.

**Figure 2:**
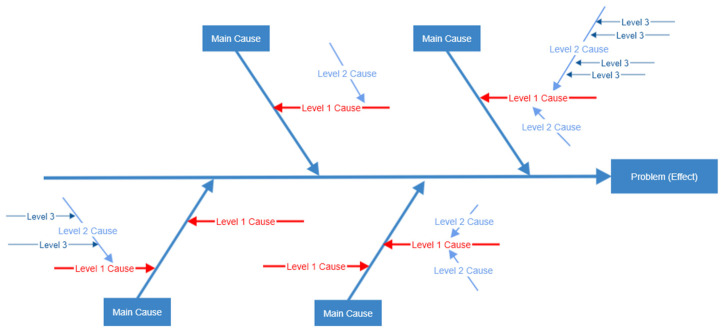
Identifying and categorizing the causal factors.

A cause-and-effect analysis was then performed on the list of factors so that they could be categorized as belonging to threat, vulnerability, or impact. This analysis used an Ishikawa diagram. Ishikawa diagrams, also known as fishbone diagrams, were developed by Professor Kaoru Ishikawa in the 1960s. Professor Ishikawa specialized in quality-management techniques. They enable potential causes of a problem to be broken down into basic elements, providing insight that may enable the problem to be resolved [17]. As Ishikawa diagrams provide a straightforward way of examining causes that create or contribute to effects, we used this approach to identify the cause of loss of confidentiality resulting from unintentional radiation. The graphical output produced by this technique also provides a holistic view of the problem under consideration. The stages involved in creating a fishbone diagram [18] are:

(i) Define the problem. State the problem or effect in a box on the right-hand side of the diagram, then draw a line to the box, creating the backbone of the fish. In this case, the effect is the loss of confidentiality from unintentional electromagnetic emanations.
(ii) Identify potential causes for the problem. The main causes are drawn as the main bones coming from the fish’s backbone. The effect will be caused by a process or function. As the risk of loss of confidentiality will be a function of threat, vulnerability, or impact, these were identified as the main causes.
(iii) Identify subcauses for the problem. Each main cause is broken down into a set of subcauses. These are drawn as bones connected to the main bones, as shown in the fishbone structure in Figure 2. The list of causal factors that had been identified during the earlier brainstorming sessions was used to subcategorize and populate the main clauses. These created three levels of detail in the resulting fishbone diagram.

(iv) Analyse potential causes. Typically, when using fishbone diagrams, a cause that is most likely to be contributing to the problem is highlighted. In this case, as the risk of loss of confidentiality can only occur if all three of the constituents of risk exist [15], i.e., threat, vulnerability and impact, we know that multiple causes must give rise to the risk. That is, there must be at least one subcause under each of the main causes of threat, vulnerability, and impact.
(v) State the identified root cause of the problem. In this case we have categorized all the identified causal factors under the main causes of threat, vulnerability, and impact to enable further analysis of the contextual and relative significance of the vulnerability causal factors.

FG-I was engaged at a workshop to produce the initial fishbone diagram. FG-II was used to validate it, by agreeing or otherwise to the categorization of the factors, at a follow up workshop, only they attended. A final workshop involving both focus groups was used to reach a consensus on the final fishbone diagram produced.

### 2.2. Interpretive Structural Modelling of the Vulnerability Factors

The technique chosen to structurally model the vulnerability factors was interpretive structural modelling (ISM). ISM was selected as it is an interactive approach that uses a group’s judgement to decide how things or elements interrelate. As a method, it helps the group to develop a deeper understanding and insight into what links the chosen elements and the nature of their relationships. ISM has been used in a range of different domains. Examples include it being used to determine the barriers to solar power installation [19], for the analysis of consumer online buying motivations [20], and to model supply chain risks [21].

**Figure 3:**
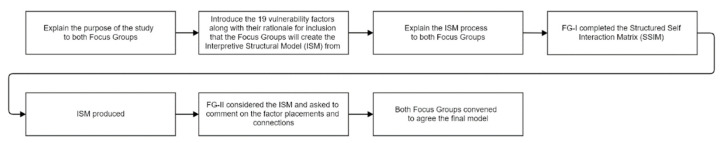
Creating the structural model from the vulnerability factors.

Both focus groups were introduced to the purpose of the study and were provided with an overview of the ISM process and their roles within it. FG-I participants were asked to complete a structural self-interaction matrix (SSIM) individually. The SSIM involves performing a pairwise comparison of the factors so that the relationships between them can be found. The returned SSIMs were combined at a workshop from which a first ISM was produced. This model was sent to FG-I for comment and the SSIM was modified as needed. This process was iterated three times until FG-I was content with the model they had produced. FG-II was presented with this model and asked to comment on the factor’s hierarchical placement and interconnections. The SSIM was changed to accommodate their views and an updated version of the model created. This updated version was sent to FG-II for comment and after two iterations was agreed. Both FG-I and FG-II were then invited to a joint workshop to consider this new version and agree on the final ISM. The approach followed is shown in Figure 3.

**Figure 4:**
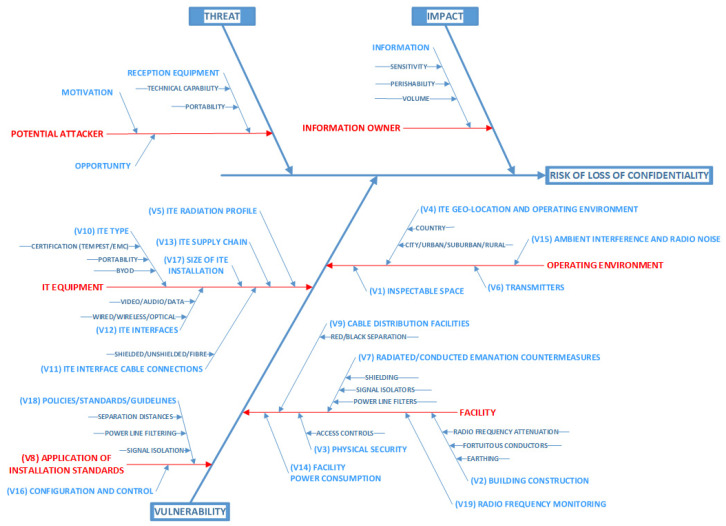
Categorization of the causal factors using the Ishikawa (fishbone) diagram.

The stages involved in the ISM process [22] are:

(i) Identify the issue to be studied. In this case, the aim is to model the vulnerability factors that RF engineers take into consideration when assessing the likelihood of unintentional electromagnetic radiation compromising office-based ITE security.
(ii) Decide on the type of ISM to be constructed. The author of [22] explains that ISM has five structures: intent, priority, attribute enhancement, process structures (sequencing) and mathematical dependence. Each of these structures will have a contextual relationship between the elements making up the ISM. Examples of contextual relationships between elements of the five structures listed in order could be ‘would help to achieve’, ‘is more important than’, ‘strongly contributes to’, ‘takes place before’, ‘maps to’. This study prioritizes the vulnerability factors whilst establishing the contribution of the factors to each other as the contextual relationship.
(iii) Select participant group and facilitator. Two focus groups of RF engineers were engaged in the study, with the lead author of this paper acting as the facilitator.
(iv) Generate the element set. The list of vulnerability factors (V1-V19) as identified in Figure 4 are used as the element set.
(v) Complete matrix of element interactions. Pairs of elements within the set were compared and a structural self-interaction matrix (SSIM) was completed based on their relationship. All FG-I RF engineers had been sent instructions explaining how to complete a SSIM, with all seven returning the completed SSIM matrix. This required them to consider 171 combinations of two factors for the vulnerability SSIM made from the 19 factors. The SSIM was completed using the following rules:
  - Vulnerability factor (i) contributes to vulnerability factor (j) (noting this as a letter V in the SSIM)
  - Vulnerability factor (j) contributes to vulnerability factor (i) (noting this as a letter A in the SSIM)
  - Both vulnerability factors contribute to each other (noting this as a letter X in the SSIM)
  - Both vulnerability factors are independent of each other (noting this as letter O in the SSIM)

The individual SSIMs were combined into a single SSIM using the majority vote as the decider for the cell value. A follow-on workshop with FG-I participants resolved any areas of disagreement. An initial reachability matrix (IRM) was created from the SSIM. The IRM has rows and columns labelled by the Factors and shows the pairwise relationship between the Factors in binary form. The rules for converting the SSIM to an IRM are:

- If the relationship between factor (i) and factor (j) is ‘V’, then the cell in the IRM labelled (i,j) is marked with a value of binary 1 and the cell labelled (j,i) is marked with a value of binary 0;
- If the relationship between factor (i) and factor (j) is ‘A’, then the cell in the IRM labelled (i,j) is marked with a value of binary 0 and the cell labelled (j,i) is marked with a value of binary 1;
- If the relationship between factor (i) and factor (j) is ‘X’, then the cell in the IRM labelled (i,j) is marked with a value of binary 1 and the cell labelled (j,i) is marked with a value of binary 1;
- If the relationship between factor (i) and factor (j) is ‘O’, then the cell in the IRM labelled (i,j) is marked with a value of binary 0 and the cell labelled (j,i) is marked with a value of binary 0.

The IRM was then checked for added inferred relationships (termed transitivity), which were added to the reachability matrix, creating a final reachability matrix (FRM). This is based on the idea that if factor X is related to factor Y and factor Y is related to factor Z, then it can be inferred that factor X will be related to factor Z. The inferred transitive relationships are shown as red cells in the FRM.

The reachability matrix was then partitioned into different hierarchical levels. The FRM rows (having a binary 1) show which other factors a factor can reach; these being termed the reachability set. The FRM columns (having a binary 1) show which factors can reach the factor in question, termed the antecedent set. An intersection set is made from the common factors of both the reachability and antecedent sets. When the reachability set is the same as the intersection set, the factor has been partitioned into a level. Once a factor has been partitioned into a level, it is removed from the reachability and antecedent sets, and the process is repeated for the remaining factors until all have eventually been assigned a level.

A canonical matrix (CM) was then produced with the factors grouped in order of the partitioned levels with the transitive links removed (i.e., the CM contains the entries from the IRM, but with the factors ordered in terms of the levels identified).

A directed graph (digraph) was then created from the CM. The 19 factors from the CM were placed at the determined levels and links drawn between them where a cell had a binary 1. Once all the cells had been examined, the digraph was complete.

(vi) Display the ISM. The digraph was then converted into an ISM by replacing the element nodes with the element names.
(vii) Discuss the structure and amend if necessary. The resulting model was then checked for conceptual consistency with both FG-I and FG-II focus groups.

## 3. Results

This section is divided into two subsections. In Section 3.1, the categorization of the causal factors resulting from the cause-and-effect analysis using the Ishikawa diagram is presented. In Section 3.2, the development of the ISM using the factors related to vulnerability is shown and a cross-impact matrix multiplication applied to classification analysis (MICMAC) is used to show the factors’ relative significance.

### 3.1. Causal Factor Categories

Risk has three components: threat, vulnerability, and impact. By mapping the 26 causal factors as subcauses to these, it was possible to show 19 of the identified factors related to vulnerability; 3 to threat; and 4 to impact. The identified factors were treated as the potential causes that could lead to the effect of loss of confidentiality. The resulting Ishikawa diagram, adapted from [10] with the vulnerability factors labelled V1–V19, is shown in Figure 4.

### 3.2. Interpretive Structural Model of the Vulnerability Factors

The research questions RQ1 and RQ2 focused on TEMPEST vulnerability. Therefore, the vulnerability causal factors V1-V19 shown in Figure 4 were used as the basis from which to build a structural model. The model and associated MICMAC analysis performed show the factors’ relationships and relative significance.

As described in Section 2, pairs of elements (i.e., vulnerability factors) within the element set were compared and a structural self-interaction matrix (SSIM) was completed based on the contextual relationship between them. The resulting SSIM is given in Table 1.

An initial reachability matrix (IRM) was created from the SSIM (Table 2). The IRM has rows and columns labelled by the vulnerability factors and shows the pairwise relationship between the factors in binary form. The rules used for converting the SSIM to an IRM were as described in Section 2.

The IRM was then checked for any inferred relationships (termed transitivity). These are added, creating a final reachability matrix (FRM). The inferred transitive relationships are shown in red in the FRM in Table 3.

From the FRM, it was then possible to develop the structural model by identifying the reachability and antecedent sets so that the factors could be levelled into a hierarchy, as shown in Table 4. Once a factor has been partitioned into a level, it is removed from the reachability and antecedent sets, and the process is repeated for the remaining factors until all have eventually been assigned a level.

The factors were then grouped in the order of the partitioned levels, with the transitive links removed, creating a canonical matrix (CM). The CM contains the entries from the IRM, but with the factors ordered in terms of the levels identified, as shown in Table 5.

**Figure 5:**
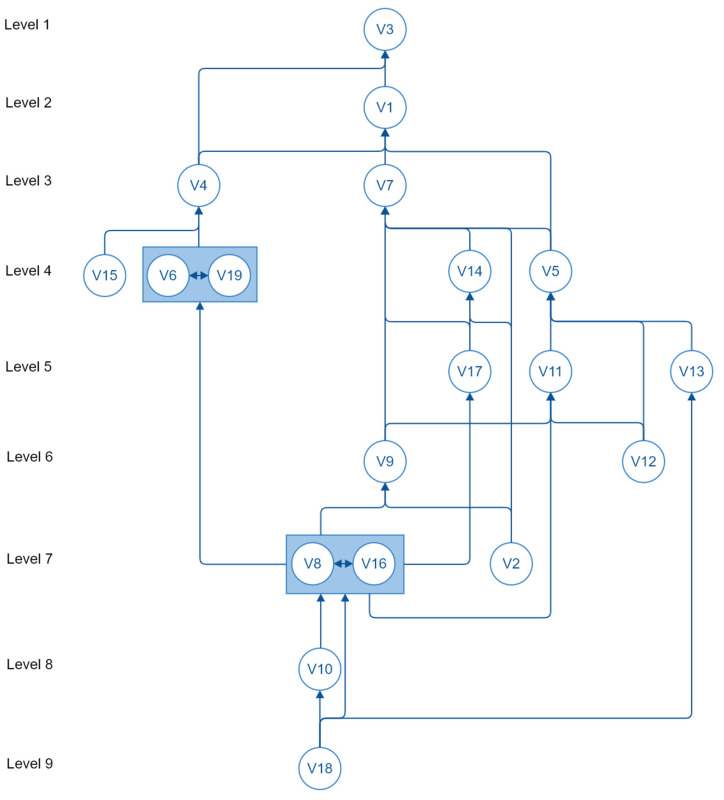
Digraph showing the relationships between the element set of vulnerability factors.

The 19 factors from the CM were placed at the determined levels. The nodes of the digraph were labelled with the vulnerability factor ID and links were drawn between them where a cell had a binary 1. This created a directed graph (digraph), shown in Figure 5, that was the basis for the structural model.

**Figure 6:**
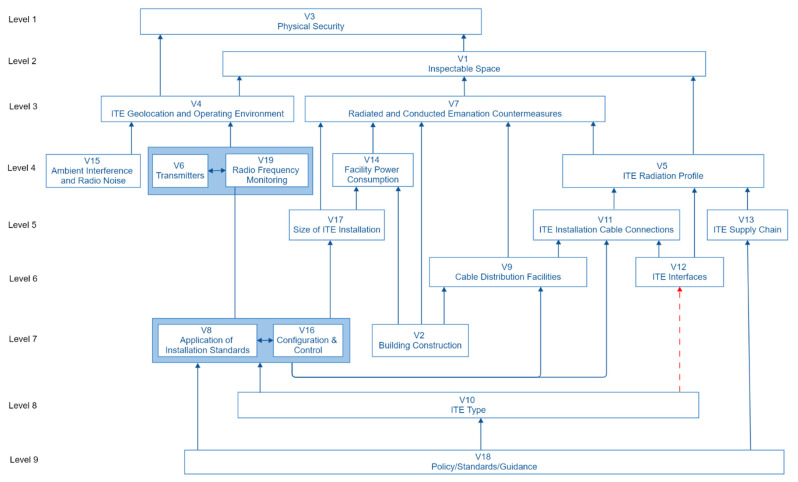
Interpretive structural model (ISM) of the vulnerability factors.

The nodes of the digraph were then named to create the model. The model was then examined by both FG-I and FG-II at the final workshop. They asked that an additional link be added between the V10 ITE Type and V12 ITE Interfaces. Both focus groups wanted to ensure that the relationship between the ITE and its interfaces was shown, as the interfaces and their associated data rates have a bearing on the TEMPEST vulnerability likelihood of the ITE and system in which it is used. This extra link is shown as a dotted line in the final ISM (Figure 6).

#### Vulnerability Factor MICMAC Analysis

To address RQ2, a cross-impact matrix multiplication applied to classification analysis (MICMAC) was performed on the vulnerability factors to determine how they influence each other. This is a two-stage process using the FRM. Firstly, the driving power of each factor is found by counting the ones in the rows, and the dependency power is determined by counting the ones in the columns. Secondly, these values are then mapped onto a grid made of four quadrants, labelled autonomous, linkage, independent (or driver) and dependent.

Autonomous factors only drive a small number of factors and are only dependent on a small number. This means they have a limited effect on the overall vulnerability likelihood. In this analysis, (V2) Building Construction, (V4) ITE Geolocation and Operating Environment, (V6) Transmitters, (V9) Cable Distribution Facilities, (V11) ITE Installation Cable Connections, (V12) ITE Interfaces, (V13) ITE Supply Chain, (V14) Facility Power Consumption, (V15) Ambient Interference and Radio Noise, (V17) Size of ITE Installation and (V19) Radio Frequency Monitoring belonged in this category. Linkage factors drive a high number of factors and are dependent on a high number of factors. This means that they can both influence and be influenced by other vulnerability factors, causing volatility within the system of interest. In this case, no linkage factors were found. Independent factors drive a high number of factors but are only dependent on a small number. This means that they will have a significant impact on the overall vulnerability likelihood. In this analysis, (V8) Application of Installation Standards, (V10) ITE Type, (V16) Configuration and Control and (V18) Policy, Standards and Guidelines belonged in this category. Dependent factors drive a small number of other factors but are dependent on a high number of factors. This means that they are strongly influenced by other vulnerability factors but do not influence others. In this analysis, (V1) Inspectable Space, (V3) Physical Security, (V5) ITE Radiation Profile and (V7) Radiated and Conducted Emanation Countermeasures belonged in this category.

**Figure 7:**
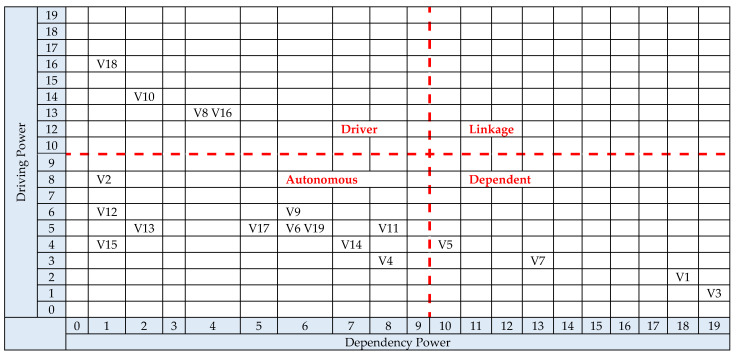
MICMAC Analysis.

The resulting analysis is shown in Figure 7. It shows that the independent (driving) factors found at the bottom of the ISM diagram in Figure 6 strongly influence the overall vulnerability likelihood as they are factors that drive through the adoption of policy and standards, equipment selection, equipment installation and change management. The dependent factors residing at the top of the ISM diagram have limited influence on other factors but show the relationship between the ITE radiation profile, the attacker proximity, and countermeasures, including physical security.

## 4. Discussion

The aims of this paper were to explore how RF engineers categorized the causal factors as either belonging to threat, vulnerability, or impact, and then to model the causal vulnerability factors so that their interpretive structure, relationships, and relative significance can be understood and shared with cyber- and information security professionals who do not possess RF experience.

To achieve this, a focus group approach was used to elicit the expert knowledge of the RF engineers engaged in the study. The use of focus groups proved useful in that it allowed for the modelling of the causal vulnerability factors and the models’ validation to be split across two different organizations. This offset the expert availability problem, as the workshops and the requirement to individually complete documentation could be more easily scheduled to meet the organizations’ work commitments.

The 26 causal factors found in [10] were validated and found to exist in the literature, e.g., [23,24,25,26,27]. However, they are not collated or categorized and their relative significance to each other is difficult to decide given the different contexts in which they are reported. The categorization of the causal factors showed that the 19 related to vulnerability focused on the ITE, the installation standards, the operating environment, and the facility in which it was deployed.

This grouping of factors aligned with the findings from [24] that showed that threat actors will apply technical capability to detect [25], then capture [14] and finally reconstitute [26] unintentional electromagnetic emanations generated from IT equipment as part of its normal operation. Risk mitigation through the application of countermeasures [23] such as signal strength reduction of the emanations (e.g., by providing separation distance and/or by architectural and equipment shielding) are deployed. The results show that the RF engineers use their expertise to concentrate on the vulnerabilities, whilst recognizing that threat capability, particularly the impact that radio receiver and antenna performance will have on the range over which emissions can be captured [27].

The resulting ISM and MICMAC analysis show the relationships between the 19 vulnerability factors and their ability to influence each other. The analysis has found four key causal factors (shown at the bottom levels of the ISM diagram in Figure 6): V18 Policy, Standards and Guidance; V10 ITE Equipment Type; V8 Application of Installation Standards; and V16 Configuration and Control. These factors drive the overall TEMPEST vulnerability likelihood. This implies that to manage TEMPEST vulnerabilities effectively relies on adopting policies, standards and guidelines, which in turn lead to the right choice of equipment. This equipment then needs to be installed correctly and configuration-controlled. Given the significance of these factors, if they are not adopted, the model shows that this will have a detrimental impact on the overall level of vulnerability likelihood. For example, if no policy is adopted, there is a risk that the ITE type selected, and installation standards applied will not be appropriate for the application. Without appropriate installation standards the change management becomes ineffective, as the standards set a baseline from which changes can be assessed. The analysis has also found four highly dependent factors (shown at the top levels of the ISM diagram in Figure 6): V3 Physical Security; V1 Inspectable Space; V7 Radiated and Conducted Emanation Countermeasures; and V5 ITE Radiation Profile. These show the relationship between the ITE’s radiation profile, and the countermeasures deployed to prevent any emanations radiating over a distance from which an attacker could benefit. It highlights the level of physical security needed as being influenced by the geolocation and operating environment of the ITE and the inspectable space (how close an attacker can get). The geolocation of the ITE may increase the risk to it, allowing potential attackers greater access to the facility in which it is housed and or greater proximity to the equipment itself. The ISM developed (Figure 6) highlights that the RF engineers are using physical security controls to keep the distance from the ITE and an attacker to the maximum. The RF Engineers are then assessing the radiation profile of the ITE against this distance, and providing that the radiation profile is less than it, they believe that the vulnerability is unlikely to be exploited. If the RF Engineers find that the radiation profile exceeds the distance that they can physically control, they will then apply countermeasures to keep the emanations within it.

Information leakage through electromagnetic emissions are now included in security management frameworks, e.g., ISO 27000, and specifically ISO 27005 [28]. However, the detail explaining these vulnerabilities and their mitigation measures is not always sufficient, requiring cybersecurity practitioners without RF experience to seek support from RF consultancy services. Additionally, it is recognized that different approaches will be used by experts and novices when processing information. This is related to the different levels of prior knowledge that each bring to a specific domain [29]. This may also extend to information security practitioners without prior knowledge of RF engineering who may not give the same consideration to the TEMPEST vulnerabilities as they would to the other cybersecurity vulnerabilities that they have more experience of.

The structural model of the causal vulnerability factors and associated MICMAC analysis produced by this study should aid cybersecurity practitioners (without RF experience) to enhance their knowledge and understanding of what is being considered as part of a TEMPEST vulnerability assessment. This will be useful when they are carrying out risk assessments that need to incorporate TEMPEST vulnerability assessments. For example, the ISM can be used to derive a series of questions that could be asked, e.g., by a cybersecurity risk manager (without RF experience) of a project to ensure that the vulnerability likelihood is being minimized. This could include, for example, focusing on an area where the project may have increased the vulnerability likelihood, e.g., by buying equipment of the wrong type or not having robust configuration and control practices, so that when a piece of equipment fails, it is replaced with the wrong type or installed without following the best installation practice. The ISM can also aid RF engineers in their professional consultancy practice by supplying a baseline model from which vulnerability assessments of office-based ITE can be made. An example would be to derive a checklist from the model. This could be used to formalize a peer review process between RF engineers to ensure they had maintained the quality of any consultancy offered.

One of the difficulties with assessing TEMPEST vulnerability likelihood is in establishing how much deviation from the ideal causes the vulnerability likelihood level to rise, and by how much. To answer this requires knowledge of the factor relationships (provided by the ISM) but also the dynamic behaviour between the factors, i.e., if one factor changes its value, what impact that has on a connected factor. This will be the focus of future work, which will use the ISM as the basis to investigate the dynamic relationships between factors, so that the vulnerability likelihood level as part of a vulnerability assessment can be quantified.

## 5. Conclusions

The output from this study provides an ISM of the causal vulnerability factors related to TEMPEST vulnerabilities of office-based ITE. The model shows the relative significance of the factors and their interrelationships. The accompanying MICMAC analysis has also found the key driving factors that affect TEMPEST vulnerability management.

The study has employed two independent focus groups of RF engineers to model the factors used in professional practice when assessing TEMPEST vulnerabilities. The model and associated MICMAC analysis can be used by cybersecurity risk managers having little or no RF experience to enhance their knowledge. This will improve their ability to manage vulnerabilities of this type by, e.g., ensuring best installation practices are followed. The ISM and related information provided by the Ishikawa diagram also provide useful artefacts as an aide-memoire for practicing RF engineers.

Future work will develop the ISM into a vulnerability assessment decision support tool, where it will be used to predict the TEMPEST vulnerability likelihood from office-based deployments of ITE. The intent is for the tool to be of use to RF engineers and to support cybersecurity practitioners who do not have RF expertise. As TEMPEST vulnerability assessment is a specialized area of RF engineering, only a limited number of RF engineers with the requisite expertise were available. This, taken into consideration with the fact that the model was developed for a specific office-based ITE deployment scenario, may impact the generality of the results. Nonetheless, the RF experts believe that the results are robust enough to provide a useful basis from which to develop a decision support tool.

## Figures and Tables

**Table 1:** Self-structured interaction matrix (SSIM).

ID	V1	V2	V3	V4	V5	V6	V7	V8	V9	V10	V11	V12	V13	V14	V15	V16	V17	V18	V19
**V1**		O	V	A	A	O	A	O	O	O	O	O	O	O	O	O	O	O	O
**V2**			O	O	O	O	V	O	V	O	O	O	O	V	O	O	O	O	O
**V3**				A	O	O	O	O	O	O	O	O	O	O	O	O	O	O	O
**V4**					O	A	O	O	O	O	O	O	O	O	A	O	O	O	A
**V5**						O	V	O	O	O	A	A	A	O	O	O	O	O	O
**V6**							O	A	O	O	O	O	O	O	O	O	O	O	X
**V7**								O	A	O	O	O	O	A	O	O	A	O	O
**V8**									V	A	V	O	O	O	O	X	V	A	O
**V9**										O	V	O	O	O	O	O	O	O	O
**V10**											O	O	O	O	O	V	O	A	O
**V11**												A	O	O	O	O	O	O	O
**V12**													O	O	O	O	O	O	O
**V13**														O	O	O	O	A	O
**V14**															O	O	A	O	O
**V15**																O	O	O	O
**V16**																	V	O	O
**V17**																		O	O
**V18**																			O
**V19**																			

**Table 2:** Initial reachability matrix (IRM).

ID	V1	V2	V3	V4	V5	V6	V7	V8	V9	V10	V11	V12	V13	V14	V15	V16	V17	V18	V19
**V1**	1	0	1	0	0	0	0	0	0	0	0	0	0	0	0	0	0	0	0
**V2**	0	1	0	0	0	0	1	0	1	0	0	0	0	1	0	0	0	0	0
**V3**	0	0	1	0	0	0	0	0	0	0	0	0	0	0	0	0	0	0	0
**V4**	1	0	1	1	0	0	0	0	0	0	0	0	0	0	0	0	0	0	0
**V5**	1	0	0	0	1	0	1	0	0	0	0	0	0	0	0	0	0	0	0
**V6**	0	0	0	1	0	1	0	0	0	0	0	0	0	0	0	0	0	0	1
**V7**	1	0	0	0	0	0	1	0	0	0	0	0	0	0	0	0	0	0	0
**V8**	0	0	0	0	0	1	0	1	1	0	1	0	0	0	0	1	1	0	0
**V9**	0	0	0	0	0	0	1	0	1	0	1	0	0	0	0	0	0	0	0
**V10**	0	0	0	0	0	0	0	1	0	1	0	0	0	0	0	1	0	0	0
**V11**	0	0	0	0	1	0	0	0	0	0	1	0	0	0	0	0	0	0	0
**V12**	0	0	0	0	1	0	0	0	0	0	1	1	0	0	0	0	0	0	0
**V13**	0	0	0	0	1	0	0	0	0	0	0	0	1	0	0	0	0	0	0
**V14**	0	0	0	0	0	0	1	0	0	0	0	0	0	1	0	0	0	0	0
**V15**	0	0	0	1	0	0	0	0	0	0	0	0	0	0	1	0	0	0	0
**V16**	0	0	0	0	0	0	0	1	0	0	0	0	0	0	0	1	1	0	0
**V17**	0	0	0	0	0	0	1	0	0	0	0	0	0	1	0	0	1	0	0
**V18**	0	0	0	0	0	0	0	1	0	1	0	0	1	0	0	0	0	1	0
**V19**	0	0	0	1	0	1	0	0	0	0	0	0	0	0	0	0	0	0	1

**Table 3:** Final reachability matrix (FRM).

ID	V1	V2	V3	V4	V5	V6	V7	V8	V9	V10	V11	V12	V13	V14	V15	V16	V17	V18	V19
**V1**	1	0	1	0	0	0	0	0	0	0	0	0	0	0	0	0	0	0	0
**V2**	1	1	1	0	1	0	1	0	1	0	1	0	0	1	0	0	0	0	0
**V3**	0	0	1	0	0	0	0	0	0	0	0	0	0	0	0	0	0	0	0
**V4**	1	0	1	1	0	0	0	0	0	0	0	0	0	0	0	0	0	0	0
**V5**	1	0	1	0	1	0	1	0	0	0	0	0	0	0	0	0	0	0	0
**V6**	1	0	1	1	0	1	0	0	0	0	0	0	0	0	0	0	0	0	1
**V7**	1	0	1	0	0	0	1	0	0	0	0	0	0	0	0	0	0	0	0
**V8**	1	0	1	1	1	1	1	1	1	0	1	0	0	1	0	1	1	0	1
**V9**	1	0	1	0	1	0	1	0	1	0	1	0	0	0	0	0	0	0	0
**V10**	1	0	1	1	1	1	1	1	1	1	1	0	0	1	0	1	1	0	1
**V11**	1	0	1	0	1	0	1	0	0	0	1	0	0	0	0	0	0	0	0
**V12**	1	0	1	0	1	0	1	0	0	0	1	1	0	0	0	0	0	0	0
**V13**	1	0	1	0	1	0	1	0	0	0	0	0	1	0	0	0	0	0	0
**V14**	1	0	1	0	0	0	1	0	0	0	0	0	0	1	0	0	0	0	0
**V15**	1	0	1	1	0	0	0	0	0	0	0	0	0	0	1	0	0	0	0
**V16**	1	0	1	1	1	1	1	1	1	0	1	0	0	1	0	1	1	0	1
**V17**	1	0	1	0	0	0	1	0	0	0	0	0	0	1	0	0	1	0	0
**V18**	1	0	1	1	1	1	1	1	1	1	1	0	1	1	0	1	1	1	1
**V19**	1	0	1	1	0	1	0	0	0	0	0	0	0	0	0	0	0	0	1

**Table 4:** Levelling into a hierarchy.

Vuln ID	Reachability Set (FRM Row)	Antecedent Set (FRM Col)	Intersection Set	Level
**1**	1, 3	1, 2, 4, 5, 6, 7, 8, 9, 10, 11, 12, 13, 14, 15, 16, 17, 18, 19	1	
**2**	1, 2, 3, 5, 7, 9, 11, 14	2	2	
**3**	3	1, 2, 3, 4, 5, 6, 7, 8, 9, 10, 11, 12, 13, 14, 15, 16, 17, 18, 19	3	**1**
**4**	1, 3, 4	4, 6, 8, 10, 15, 16, 18, 19	4	
**5**	1, 3, 5, 7	2, 5, 8, 9, 10, 11, 12, 13, 16, 18	5	
**6**	1, 3, 4, 6, 19	6, 8, 10, 16, 18, 19	6, 19	
**7**	1, 3, 7	2, 5, 7, 8, 9, 10, 11, 12, 13, 14, 16, 17, 18	7	
**8**	1, 3, 4, 5, 6, 7, 8, 9, 11, 14, 16, 17, 18	8, 10, 16, 18	8, 16, 18	
**9**	1, 3, 5, 7, 9, 11	2, 8, 9, 10, 16, 18	9	
**10**	1, 3, 4, 5, 6, 7, 8, 9, 10, 11, 14, 16, 17, 19	10, 18	10	
**11**	1, 3, 5, 7, 11	2, 8, 9, 10, 11, 12, 16, 18	11	
**12**	1, 3, 5, 7, 11, 12	12	12	
**13**	1, 3, 5, 7, 13	13, 18	13	
**14**	1, 3, 7, 14	2, 8, 10, 14, 16, 17, 18	14	
**15**	1, 3, 4, 15	15	15	
**16**	1, 3, 4, 5, 6, 7, 8, 9, 11, 14, 16, 17, 19	8, 10, 16, 18	8, 16	
**17**	1, 3, 7, 14, 17	8, 10, 16, 17, 18	17	
**18**	1, 3, 4, 5, 6, 7, 8, 9, 10, 11, 13, 14, 16, 17, 18, 19	18	18	
**19**	1, 3, 4, 6, 19	6, 8, 10, 16, 18, 19	6, 19	
**Remove Factor V3**				
**1**	1	1, 2, 4, 5, 6, 7, 8, 9, 10, 11, 12, 13, 14, 15, 16, 17, 18, 19	1	**2**
**2**	1, 2, 5, 7, 9, 11, 14	2	2	
**4**	1, 4	4, 6, 8, 10, 15, 16, 18, 19	4	
**5**	1, 5, 7	2, 5, 8, 9, 10, 11, 12, 13, 16, 18	5	
**6**	1, 4, 6, 19	6, 8, 10, 16, 18, 19	6, 19	
**7**	1, 7	2, 5, 7, 8, 9, 10, 11, 12, 13, 14, 16, 17, 18	7	
**8**	1, 4, 5, 6, 7, 8, 9, 11, 14, 16, 17, 18	8, 10, 16, 18	8, 16, 18	
**9**	1, 5, 7, 9, 11	2, 8, 9, 10, 16, 18	9	
**10**	1, 4, 5, 6, 7, 8, 9, 10, 11, 14, 16, 17, 19	10, 18	10	
**11**	1, 5, 7, 11	2, 8, 9, 10, 11, 12, 16, 18	11	
**12**	1, 5, 7, 11, 12	12	12	
**13**	1, 5, 7, 13	13, 18	13	
**14**	1, 7, 14	2, 8, 10, 14, 16, 17, 18	14	
**15**	1, 4, 15	15	15	
**16**	1, 4, 5, 6, 7, 8, 9, 11, 14, 16, 17, 19	8, 10, 16, 18	8, 16	
**17**	1, 7, 14, 17	8, 10, 16, 17, 18	17	
**18**	1, 4, 5, 6, 7, 8, 9, 10, 11, 13, 14, 16, 17, 18, 19	18	18	
**19**	1, 4, 6, 19	6, 8, 10, 16, 18, 19	6, 19	
**Remove Factor V1**				
**2**	2, 5, 7, 9, 11, 14	2	2	
**4**	4	4, 6, 8, 10, 15, 16, 18, 19	4	**3**
**5**	5, 7	2, 5, 8, 9, 10, 11, 12, 13, 16, 18	5	
**6**	4, 6, 19	6, 8, 10, 16, 18, 19	6, 19	
**7**	7	2, 5, 7, 8, 9, 10, 11, 12, 13, 14, 16, 17, 18	7	**3**
**8**	4, 5, 6, 7, 8, 9, 11, 14, 16, 17, 18	8, 10, 16, 18	8, 16, 18	
**9**	5, 7, 9, 11	2, 8, 9, 10, 16, 18	9	
**10**	4, 5, 6, 7, 8, 9, 10, 11, 14, 16, 17, 19	10, 18	10	
**11**	5, 7, 11	2, 8, 9, 10, 11, 12, 16, 18	11	
**12**	5, 7, 11, 12	12	12	
**13**	5, 7, 13	13, 18	13	
**14**	7, 14	2, 8, 10, 14, 16, 17, 18	14	
**15**	4, 15	15	15	
**16**	4, 5, 6, 7, 8, 9, 11, 14, 16, 17, 19	8, 10, 16, 18	8, 16	
**17**	7, 14, 17	8, 10, 16, 17, 18	17	
**18**	4, 5, 6, 7, 8, 9, 10, 11, 13, 14, 16, 17, 18, 19	18	18	
**19**	4, 6, 19	6, 8, 10, 16, 18, 19	6, 19	
**Remove Factors V4 and V7**				
**2**	2, 5, 9, 11, 14	2	2	
**5**	5	2, 5, 8, 9, 10, 11, 12, 13, 16, 18	5	**4**
**6**	6, 19	6, 8, 10, 16, 18, 19	6, 19	**4**
**8**	5, 6, 8, 9, 11, 14, 16, 17, 18	8, 10, 16, 18	8, 16, 18	
**9**	5, 9, 11	2, 8, 9, 10, 16, 18	9	
**10**	5, 6, 8, 9, 10, 11, 14, 16, 17, 19	10, 18	10	
**11**	5, 11	2, 8, 9, 10, 11, 12, 16, 18	11	
**12**	5, 11, 12	12	12	
**13**	5, 13	13, 18	13	
**14**	14	2, 8, 10, 14, 16, 17, 18	14	**4**
**15**	15	15	15	**4**
**16**	5, 6, 8, 9, 11, 14, 16, 17, 19	8, 10, 16, 18	8, 16	
**17**	14, 17	8, 10, 16, 17, 18	17	
**18**	5, 6, 8, 9, 10, 11, 13, 14, 16, 17, 18, 19	18	18	
**19**	6, 19	6, 8, 10, 16, 18, 19	6, 19	**4**
**Remove Factors V5, V6, V14, V15 and V19**				
**2**	2, 9, 11	2	2	
**8**	8, 9, 11, 16, 17, 18	8, 10, 16, 18	8, 16, 18	
**9**	9, 11	2, 8, 9, 10, 16, 18	9	
**10**	8, 9, 10, 11, 16, 17	10, 18	10	
**11**	11	2, 8, 9, 10, 11, 12, 16, 18	11	**5**
**12**	11, 12	12	12	
**13**	13	13, 18	13	**5**
**16**	8, 9, 11, 16, 17	8, 10, 16, 18	8, 16	
**17**	17	8, 10, 16, 17, 18	17	**5**
**18**	8, 9, 10, 11, 13, 16, 17, 18	18	18	
**Remove Factors V11, V13 and V17**				
**2**	2, 9	2	2	
**8**	8, 9, 16, 18	8, 10, 16, 18	8, 16, 18	
**9**	9	2, 8, 9, 10, 16, 18	9	**6**
**10**	8, 9, 10, 16	10, 18	10	
**12**	12	12	12	**6**
**16**	8, 9, 16	8, 10, 16, 18	8, 16	
**18**	8, 9, 10, 16, 18	18	18	
**Remove Factors V9 and V12**				
**2**	2	2	2	**7**
**8**	8, 16, 18	8, 10, 16, 18	8, 16, 18	**7**
**10**	8, 10, 16	10, 18	10	
**16**	8, 16	8, 10, 16, 18	8, 16	**7**
**18**	8, 10, 16, 18	18	18	
**Remove Factors V2, V8 and V16**				
**10**	10	10, 18	10	**8**
**18**	10, 18	18	18	
**Remove Factor V10**				
**18**	18	18	18	**9**

**Table 5:** Canonical matrix (CM).

ID	V3	V1	V4	V7	V5	V6	V14	V15	V19	V11	V13	V17	V9	V12	V2	V8	V16	V10	V18
**V3**	1	0	0	0	0	0	0	0	0	0	0	0	0	0	0	0	0	0	0
**V1**	1	1	0	0	0	0	0	0	0	0	0	0	0	0	0	0	0	0	0
**V4**	1	1	1	0	0	0	0	0	0	0	0	0	0	0	0	0	0	0	0
**V7**	0	1	0	1	0	0	0	0	0	0	0	0	0	0	0	0	0	0	0
**V5**	0	1	0	1	1	0	0	0	0	0	0	0	0	0	0	0	0	0	0
**V6**	0	0	1	0	0	1	0	0	1	0	0	0	0	0	0	0	0	0	0
**V14**	0	0	0	1	0	0	1	0	0	0	0	0	0	0	0	0	0	0	0
**V15**	0	0	1	0	0	0	0	1	0	0	0	0	0	0	0	0	0	0	0
**V19**	0	0	1	0	0	1	0	0	1	0	0	0	0	0	0	0	0	0	0
**V11**	0	0	0	0	1	0	0	0	0	1	0	0	0	0	0	0	0	0	0
**V13**	0	0	0	0	1	0	0	0	0	0	1	0	0	0	0	0	0	0	0
**V17**	0	0	0	1	0	0	1	0	0	0	0	1	0	0	0	0	0	0	0
**V9**	0	0	0	1	0	0	0	0	0	1	0	0	1	0	0	0	0	0	0
**V12**	0	0	0	0	1	0	0	0	0	1	0	0	0	1	0	0	0	0	0
**V2**	0	0	0	1	0	0	1	0	0	0	0	0	1	0	1	0	0	0	0
**V8**	0	0	0	0	0	1	0	0	0	1	0	1	1	0	0	1	1	0	0
**V16**	0	0	0	0	0	0	0	0	0	0	0	1	0	0	0	1	1	0	0
**V10**	0	0	0	0	0	0	0	0	0	0	0	0	0	0	0	1	1	1	0
**V18**	0	0	0	0	0	0	0	0	0	0	1	0	0	0	0	1	0	1	1

## Data Availability

Not applicable.
